# An eHealth App (CAPABLE) Providing Symptom Monitoring, Well-Being Interventions, and Educational Material for Patients With Melanoma Treated With Immune Checkpoint Inhibitors: Protocol for an Exploratory Intervention Trial

**DOI:** 10.2196/49252

**Published:** 2023-10-11

**Authors:** Itske Fraterman, Barbara M Wollersheim, Valentina Tibollo, Savannah Lucia Catherina Glaser, Stephanie Medlock, Ronald Cornet, Matteo Gabetta, Vitali Gisko, Ella Barkan, Nicola di Flora, David Glasspool, Alexandra Kogan, Giordano Lanzola, Roy Leizer, Henk Mallo, Manuel Ottaviano, Mor Peleg, Lonneke V van de Poll-Franse, Nicole Veggiotti, Konrad Śniatała, Szymon Wilk, Enea Parimbelli, Silvana Quaglini, Mimma Rizzo, Laura Deborah Locati, Annelies Boekhout, Lucia Sacchi, Sofie Wilgenhof

**Affiliations:** 1 Department of Psychosocial Research and Epidemiology The Netherlands Cancer Institute Amsterdam Netherlands; 2 Laboratory of Informatics and Systems Engineering for Clinical Research Istituti Clinici Scientifici Maugeri SpA SB IRCCS Pavia Italy; 3 Medical Informatics Amsterdam UMC University of Amsterdam Amsterdam Netherlands; 4 Methodology Amsterdam Public Health Research Institute Amsterdam Netherlands; 5 Aging and Later Life Amsterdam Public Health Research Institute Amsterdam Netherlands; 6 BIOMERIS SRL Pavia Italy; 7 Department of Electrical, Computer and Biomedical Engineering University of Pavia Pavia Italy; 8 BITSENS JSC Vilnius Lithuania; 9 Department of Artificial Intelligence for Accelerated Healthcare and Life Sciences Discovery IBM Research IBM R&D Laboratories Haifa Israel; 10 Associazione Italiana Malati di Cancro Rome Italy; 11 Deontics Ltd London United Kingdom; 12 Department of Information Systems University of Haifa Haifa Israel; 13 Department of Medical Oncology Netherlands Cancer Institute–Antoni van Leeuwenhoek Amsterdam Netherlands; 14 Life Supporting Technologies Universidad Politécnica de Madrid Madrid Spain; 15 Department of Research and Development Netherlands Comprehensive Cancer Organization Utrecht Netherlands; 16 Department of Medical and Clinical Psychology Center of Research on Psychological and Somatic Disorders (CoRPS) Tilburg University Tilburg Netherlands; 17 Institute of Computing Science Poznan University of Technology Poznan Poland; 18 Division of Medical Oncology Azienda Ospedaliero Universitaria Consorziale Policlinico di Bari Bari Italy; 19 Department of Internal Medicine and Medical Therapy University of Pavia Pavia Italy; 20 Medical Oncology Unit, Istituti Clinici Scientifici Maugeri IRCCS Pavia Italy

**Keywords:** eHealth, melanoma, fatigue, quality of life, intervention, pilot study, QoL, cancer, oncology, HRQoL, fatigue, symptom, symptoms, monitoring, adoption, acceptance, patient education, digital health, immune checkpoint inhibitors, immunotherapy

## Abstract

**Background:**

Since treatment with immune checkpoint inhibitors (ICIs) is becoming standard therapy for patients with high-risk and advanced melanoma, an increasing number of patients experience treatment-related adverse events such as fatigue. Until now, studies have demonstrated the benefits of using eHealth tools to provide either symptom monitoring or interventions to reduce treatment-related symptoms such as fatigue. However, an eHealth tool that facilitates the combination of both symptom monitoring and symptom management in patients with melanoma treated with ICIs is still needed.

**Objective:**

In this pilot study, we will explore the use of the CAPABLE (Cancer Patients Better Life Experience) app in providing symptom monitoring, education, and well-being interventions on health-related quality of life (HRQoL) outcomes such as fatigue and physical functioning, as well as patients’ acceptance and usability of using CAPABLE.

**Methods:**

This prospective, exploratory pilot study will examine changes in fatigue over time in 36 patients with stage III or IV melanoma during treatment with ICI using CAPABLE (a smartphone app and multisensory smartwatch). This cohort will be compared to a prospectively collected cohort of patients with melanoma treated with standard ICI therapy. CAPABLE will be used for a minimum of 3 and a maximum of 6 months. The primary endpoint in this study is the change in fatigue between baseline and 3 and 6 months after the start of treatment. Secondary end points include HRQoL outcomes, usability, and feasibility parameters.

**Results:**

Study inclusion started in April 2023 and is currently ongoing.

**Conclusions:**

This pilot study will explore the effect, usability, and feasibility of CAPABLE in patients with melanoma during treatment with ICI. Adding the CAPABLE system to active treatment is hypothesized to decrease fatigue in patients with high-risk and advanced melanoma during treatment with ICIs compared to a control group receiving standard care. The Medical Ethics Committee NedMec (Amsterdam, The Netherlands) granted ethical approval for this study (reference number 22-981/NL81970.000.22).

**Trial Registration:**

ClinicalTrials.gov NCT05827289; https://clinicaltrials.gov/study/NCT05827289

**International Registered Report Identifier (IRRID):**

DERR1-10.2196/49252

## Introduction

In recent years, the introduction of immunotherapy with immune checkpoint inhibitors (ICIs) has significantly improved the clinical outcome of patients with melanoma and has become the standard of care [1-4]. However, treatment with ICIs is associated with short- and long-term immune-related adverse events (AEs) [5-7], most commonly fatigue [7,8], and a deteriorated health-related quality of life (HRQoL) [6,9,10]. Some studies have reported that inadequate symptom monitoring and reporting (eg, under detection) can lead to worsening of AEs and more frequent emergency department visits and hospitalizations [11-13]. Therefore, enhanced symptom monitoring in the general cancer population as well as in this specific patient population is necessary. Furthermore, enhanced symptom monitoring is associated with improved clinical outcomes (eg, survival and adverse event management) and improved HRQoL in patients with cancer treated with chemotherapy [14-17].

One approach to monitoring symptoms and potentially enhancing patient-centered care can be the periodic collection of symptom information through patient-reported outcome measures by using eHealth tools [18,19]. Results from several studies show that web-based symptom monitoring tools are also beneficial for patients with cancer receiving ICIs [20-22]. However, in one study specifically, the use of an electronic patient-reported outcomes tool alone in patients with melanoma treated with ICIs could not reduce the number of severe AEs. Despite there being no impact on reducing the number of severe AEs, the intervention group had significantly better HRQoL outcomes compared to the control group as measured by the utility scores of the EQ-5D (*P*=.05) [22,23]. Furthermore, home monitoring by biometrical sensors could have the potential to detect symptoms (complementary) and monitor physical activity in outpatient oncology settings, although this has not been extensively researched [24,25].

As cancer-related fatigue (CRF) is the most common symptom experienced by patients with melanoma during and after ICIs [8] and a common problem in patients with cancer in general [26,27], much effort has been made to develop interventions to address this, as CRF negatively impacts HRQoL [26,28]. Current recommendations for all patients with cancer suggest encouraging physical exercise and providing psychosocial interventions and psychoeducation [28]. Multiple studies investigating these interventions have shown a decrease in fatigue and an increase in HRQoL [29-34]. Furthermore, mindfulness-based clinical interventions and yoga appear to be feasible interventions, and beneficial effects have been reported on several physical and psychosocial symptoms in patients with cancer, such as patients’ psychosocial adjustment to their disease and improvement of biomarkers of stress, inflammation, and immune function [35-37]. Encouraging physical exercise, psychoeducation, mindfulness-based interventions, and yoga have therefore been included in web-based programs and eHealth apps targeting CRF, with promising results [38-41].

eHealth apps may also contribute to addressing the unmet care and information needs of patients [42,43]. Patients with melanoma during and after targeted therapies or ICIs have extensive needs for supportive care, information provision, and symptom management, specifically with regard to clinical- and self-management topics, CRF management, and available supportive care services [43]. By providing remote monitoring and information provision through an eHealth tool, patients believe this will positively affect their HRQoL and symptom burden [43].

This study will investigate the extensively pretested (manuscript in preparation) CAPABLE (Cancer Patients Better Life Experience) eHealth app providing information and supportive care, symptom monitoring, and well-being interventions (as part of the European Union’s Horizon 2020 research and innovation program under grant agreement number 875052). CAPABLE functionalities are accessible for patients through a mobile app, connected to a smartwatch, and available for health care professionals (HCPs) through a web portal. In this pilot study, we will explore the effect of using CAPABLE on HRQoL outcomes such as fatigue and physical functioning and patients’ acceptability of using a system like CAPABLE. The results of this pilot trial will be compared with a historical prospective cohort of patients with the same characteristics but without CAPABLE. We hypothesize that patients using the CAPABLE app will have a reduced increase in fatigue in the first 3-6 months of treatment compared to patients in the control cohort.

## Methods

### Overview

The CAPABLE study is a prospective, exploratory pilot study in which we compare an exploratory cohort that receives the CAPABLE smartphone app and a multisensory smartwatch (intervention) to a historical prospective cohort that did not receive the CAPABLE app and smartwatch (control group). The control group (NL75996.031.20) consists of patients with the same inclusion criteria and self-reported data collection as the intervention group but receives standard care without the CAPABLE app (eg, the patient calls the hospital when a symptom is experienced and has a blood analysis and follow-up appointment with a nurse practitioner before every ICI infusion). The initial plan of the CAPABLE consortium was to perform a randomized trial, but due to time constraints in the project, development issues, and the COVID-19 pandemic, we were not able to set up a randomized study and include the needed number of patients because of the limited numbers of patients with melanoma in the restricted and foreseen time period. No data safety monitoring board is in place due to the low risk of the intervention, as assessed by the internal data security team. The results of the study will be published in international peer-reviewed journals.

### Study Population

This study aims to include 36 patients with histologically confirmed stage III or IV melanoma eligible for starting treatment with ICIs (anti-PD1 or anti-CTLA4) according to standard clinical practice. In order to be eligible to participate in this study, a subject must be >18 years of age, have a sufficient understanding of the Dutch language, and be able to use a smartphone. Patients are excluded if they are included in an experimental clinical trial.

### Recruitment and Data Collection

**Figure 1:**
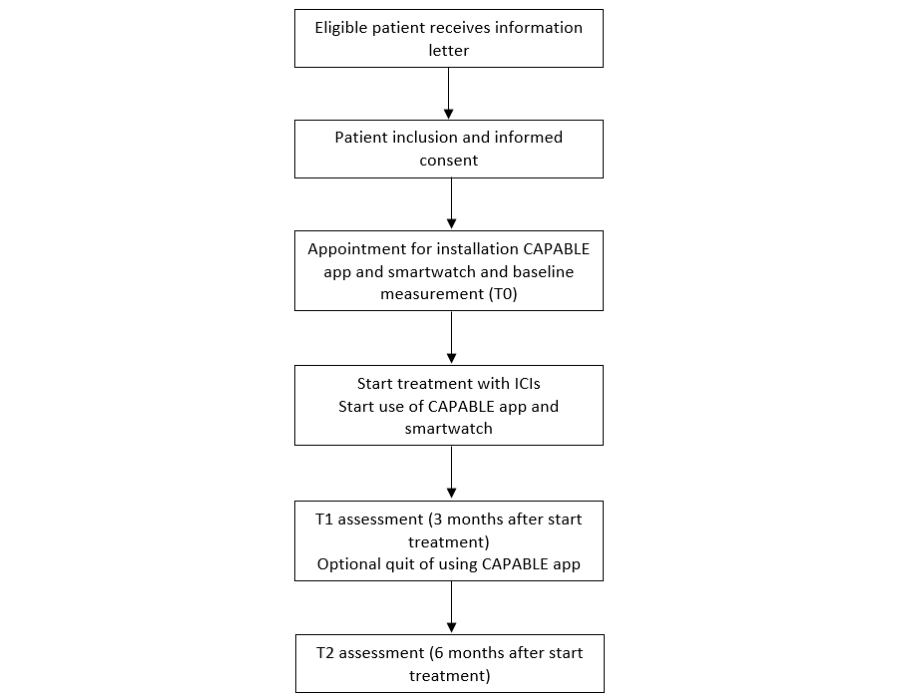
CAPABLE study flowchart. CAPABLE: Cancer Patients Better Life Experience; ICI: immune checkpoint inhibitor.

Patients will be recruited over a period of 6 months at the Netherlands Cancer Institute (NKI) in Amsterdam, a comprehensive cancer center. Eligible patients will be invited to participate in the study by their treating physician approximately 2 weeks before the start of treatment during an outpatient visit. Patients will then receive an information letter about the study from the physician or coordinating researcher. After receiving written informed consent from the patient, the patient will be enrolled in the CAPABLE system. Consenting patients will have an intake appointment with the research team before their first ICI infusion to install and set up the CAPABLE app and smartwatch. Furthermore, patients will receive a verbal explanation and an instruction manual on how to use the CAPABLE app. Patients will be asked to use the smartphone app for a minimum of 3 to a maximum of 6 months after the start of ICI treatment. In case the patient is not using the system or is not wearing the smartwatch for at least 2 weeks, the research team will contact the patient to assess possible technical issues or drop out for other reasons. Research data are collected at the intake appointment at baseline (T0), 3 (T1), and 6 months (T2) after the start of treatment (Figure 1). Data will be extracted from medical records and validated questionnaires. The patient will receive a link to a digital platform (ALEA, FormVision) through email to fill out the questionnaires. Reminders will be sent to patients’ emails if the questionnaires are not completed after 2 weeks or if they are asked to fill them out during a follow-up visit in the hospital. Clinical data (eg, staging, treatment details, and demographics) will be extracted from the medical record during the study. Data will be coded and only accessible by the NKI research team on a secured server. Patient recruitment and data collection started in April 2023 and are currently ongoing.

### CAPABLE Intervention

#### Overview

**Figure 2:**
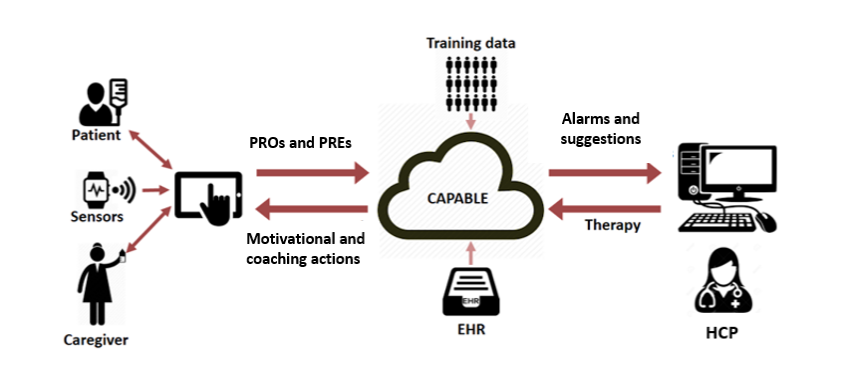
Overall structure of the CAPABLE system; the CAPABLE backend (depicted as a cloud) is shown in the middle and connects to the mobile app on the patient side and the web-based dashboard on the HCP’s side. CAPABLE: Cancer Patients Better Life Experience; EHR: electronic health record; HCP: health care professional; PRE: patient-reported experience; PRO: patient-reported outcome.

CAPABLE is an eHealth tool consisting of front end and backend components (mobile app and smartwatch for patients, web-based dashboard for HCPs) that users interact with and that provide functionality related to decision support and coaching. CAPABLE monitors treatment-related symptoms and well-being and provides patients with extensive educational material and nonpharmacological evidence-based interventions called Virtual Capsules (VCs). VCs are developed using Fogg behavioral model [44] to help patients turn the proposed interventions into healthy habits. CAPABLE consists of several components that run on the backend of the system. Patient-reported information is made available to HCPs through the physician web dashboard, which can only be accessed by users in the private network of the hospital using personal credentials to login. Around 15 HCPs (medical oncologists and nurse practitioners in training) involved in the treatment of enrolled patients have access to the CAPABLE dashboard. Compared to usual clinical care, the CAPABLE dashboard is checked daily by the HCPs to see new alerts for entered symptoms or questionnaires. Besides this, existing care structures stay in place and symptom management is the same as in usual care. Figure 2 shows the overall structure of the CAPABLE system. CAPABLE will be connected 1-sided to the electronic health record (EHR), meaning the CAPABLE system can extract EHR data but cannot write back data into the EHR. Each component of the CAPABLE system will be deployed on a Virtual Machine provided by the hospital. Access to the Virtual Machines for the CAPABLE software developers will be possible through dedicated virtual private networks set up by NKI’s information technology staff.

During the use of the CAPABLE system, the patient will be able to perform the following set of activities through the smartphone app:

- Report symptoms using a self-developed 130-item list derived from and based on the National Cancer Institute’s (NCI) Common Terminology Criteria for Adverse Events (CTCAE) version 5 [45] and the Patient-Reported Outcome version of the CTCAE (PRO-CTCAE) standard term lists [46], which are considered self-reportable by the CAPABLE clinical expert team;
- Tracking and viewing activities and vital signs when wearing the smartwatch;
- Answer to periodic questionnaires concerning mental well-being;
- Work on well-being goals (eg, sleep improvement, improving physical or mental well-being, accepting the cancer journey) through interventions delivered by VCs;
- Access evidence-based educational material regarding disease, treatments, side effects, lifestyle, well-being interventions, supportive care, and links to peer support.

#### Symptom Monitoring

The CAPABLE smartphone app facilitates patient symptom reporting at any moment in time, based on when a symptom occurs for this patient. Patients can access their app, choose a symptom from the symptom list, and select the symptom description that matches their experience of the symptom. For each symptom, the patient can select the symptom description that matches the patient’s experience of the symptom in terms of severity and impact on activities of daily living. These descriptions allow the CAPABLE app to get specific information about the experienced symptom and give appropriate feedback and advice to the patient and HCPs. Since the NCI’s CTCAE grading system is intended for use by HCPs, it cannot be directly exploited as a symptom reporting system for patients. Furthermore, NCI’s PRO-CTCAE list was not considered fully suitable for the purposes of CAPABLE. Therefore, we created a symptom list based on the full CTCAE list, from which we removed AEs that were not detectable by a patient or his or her caregiver, used a priority scoring system of involved HCPs, and made the remaining symptom list patient-friendly, when possible exploiting the PRO-CTCAE descriptions. The descriptions were constructed by following the structure of the PRO-CTCAE monitoring, namely, defining the symptom severity as “mild,” “moderate,” “severe,” or “very severe” and defining interference with usual or daily activities as “a little bit,” “somewhat,” “quite a bit,” and “very much.” In addition, the CTCAE terms of the symptoms and information from the CTCAE grades were incorporated in the symptom description to help the patient distinguish between a “mild,” “moderate,” “severe,” or “very severe” symptom. Symptoms can be mapped to grades in CTCAE version 5.

After a patient reports a symptom, the CAPABLE clinical decision support system provides feedback that is based on implemented computer-interpretable clinical guidelines for immunotherapy toxicity management (based on CTCAE version 5) and contact levels defined by participating clinicians. For example, the CAPABLE system can assign self-care instructions to the patient after reporting low-grade skin toxicity and will also send an alert to the HCP [5]. After clinical assessment of the entered symptom, the HCP can further decide how to manage the symptom [5]. The HCP will be able to report symptoms after seeing the patient during an outpatient visit. The list of symptoms provided to HCPs on the dashboard includes the full list of CTCAE terms and the related grading. Furthermore, the HCP receives specific recommendations related to the management of the reported symptoms. The dashboard is checked every workday by the HCPs. During the weekends, patients are instructed to call the hospital in cases of severe symptoms.

#### Monitoring Mental Well-Being

**Figure 3:**
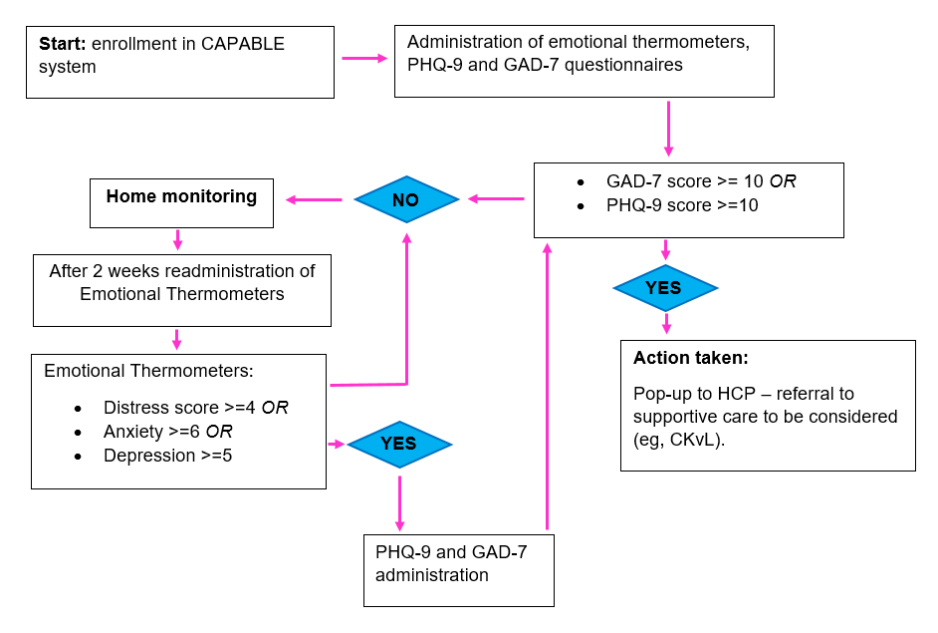
Psychological workflow in the CAPABLE system. CAPABLE: Cancer Patients Better Life Experience; CKvL: Center for Quality of Life; GAD-7: Generalized Anxiety Disorder-7; HCP: health care professional; PHQ-9: Patient Health Questionnaire-9.

Besides the symptoms related to immunotherapy, the CAPABLE app also facilitates home monitoring of mental well-being through Distress (Emotional) Thermometers [47] and 2 protocolled and validated questionnaires: the Patient Health Questionnaire-9 (PHQ-9) [48] and Generalized Anxiety Disorder-7 (GAD-7) scale [49]. The management of the questionnaire outcomes is also performed by the CAPABLE clinical decision support system and is based on clinical cutoffs [49,50]. The management strategies have been thoroughly discussed with support consultants in the hospital and will allow a possible earlier referral to supportive care in the Center for Quality of Life in the hospital for further follow-up. The complete workflow for psychological follow-up is shown in Figure 3.

#### Educational Material

In a previous study, we identified unmet information needs in patients with melanoma treated with ICIs and how this could be integrated into an eHealth tool [43]. Based on this study, we have developed a content list of information that should be provided in the CAPABLE app as educational material [43]. In more detail, the content of the educational material in the CAPABLE app consists of extensive information on the following topics:

- General melanoma content (diagnosis, diagnostic methods, causes, and prevention)
- Treatment with ICIs (information on the different immunotherapies)
- Side effects of ICIs (detailed descriptions per side effect)
- Experiences from fellow patients and peer support
- Supportive care options
- Nutrition
- Work and cancer
- Intimacy, sexuality, and cancer
- Adolescence and young adult care
- Other relevant topics (with links to external resources)

The content is created from evidence-based information leaflets already offered by the hospital and the European Society for Medical Oncology. Medical specialists and supportive care consultants from the hospital checked all content that was embedded in the CAPABLE mobile app.

#### Well-Being Interventions

CAPABLE offers patients a set of functionalities to set goals to work on various aspects of mental, physical, and social well-being at home. The interventions added to the CAPABLE system are nonpharmacological, evidence-based interventions that have been shown to improve the mental and social well-being of patients with cancer, as well as other stress-related conditions such as sleep problems and fatigue. The process works by setting specific goals for the patients (eg, sleep improvement) and selecting specific interventions to achieve those goals. Goal setting and intervention selection will be done together by the patient and HCP during the intake appointment, which facilitates shared decision-making. Interventions can serve multiple goals at once. It is always possible for the HCP to contraindicate an intervention for the patient if the conditions to perform the related activity are not met. The interventions available in the CAPABLE app and their related goals are listed below:

- Thai Chi (goals: improve sleep and insomnia, mental well-being, physical well-being) [51]
- Yoga Hatha or Nidra (goals: improve sleep and insomnia, mental well-being, physical well-being) [28]
- Walking in nature (goals: improve sleep and insomnia, mental well-being, physical well-being) [28]
- Breathing exercises and mindfulness (goals: improve sleep and insomnia, mental well-being)
- Imagery meditation (goals: improve sleep and insomnia, mental well-being) [52]
- Photo diary (goals: mental well-being, accepting the cancer journey) [53]
- Gratitude journal (goals: mental well-being, accepting the cancer journey) [54,55]

#### Smartwatch

Patients will also be provided with a smartwatch (the ASUS VivoWatch 5 HC-B05), which will be set up during enrollment. To ensure proper communication with the ASUS Life cloud platform (OmniCare), an additional app (OmniCare Hub, provided by ASUS Life) needs to be installed on the patients’ smartphones. The smartwatch will collect data on heart rate and blood pressure, sleep (stages, hours, and performance), physical activity, and stress. However, data from the smartwatch is treated as ancillary data and will not be used for symptom monitoring or diagnosis.

### Study End Points

#### Primary Outcome

The primary end point of this study is the change in fatigue between baseline and 3 and 6 months, as measured by The European Organization for Research and Treatment of Cancer (EORTC) Quality of Life Questionnaire-Core 30 (QLQ-C30) [56]. The changes in fatigue over time in the intervention cohort will be compared to the changes in fatigue over time in the control cohort. Fatigue is constructed out of 3 questions: “Did you need to rest?” “Have you felt weak?” “Were you tired?” Responses range from 1 (not at all) to 4 (very much) and are linearly transformed into a fatigue scale ranging from 0 to 100, with higher scores representing more experienced fatigue.

#### Secondary Outcomes

Secondary end points of this study are changes in physical symptoms and functioning scales [56], melanoma-specific HRQoL [57], utilities used for establishing quality-adjusted life years [58], anxiety and depression [59], patient-reported immune-related AEs and information needs [60] (Table 1). Furthermore, we will assess feasibility and patient-reported usability outcomes [61-63] at different time points (Table 1). Additionally, component-specific technical end points (clicks or completion of content in the patient app, accepted recommendations or clicks in the HCP dashboard), referrals to additional care, and the number of emergency visits will be measured.

HCPs using the CAPABLE system will retrieve extensive self-developed usability questionnaires consisting of questions about expectations, overall satisfaction, perceived impact, technical errors, ease and usefulness, efforts and resources, and the validated System Usability Scale (SUS) [64]. HCPs will receive these questionnaires after the first patient enrollment, after 1 month of using the system, and at the end of the study. They are covered in a different approved study protocol (P23CAP; IRBd23-012).

**Table 1:** Study end points and corresponding questionnaires.

Description of outcome			Assessment	T0	T1	T2
Sociodemographic data, disease, and treatment characteristics			Sociodemographic and clinical data will be abstracted from medical records or reported by the patient through the CAPABLE application^a^	✓	✓	✓
**Primary end point**						
	**PROMs^b^**					
		Fatigue	EORTC QLQ-C30 [56]^c,d^	✓	✓	✓
**Secondary end points**						
	**PROMs**					
		Physical-, social-, emotional-, cognitive- and role functioning, symptoms, and overall HRQoL^e^ (summary score)	EORTC QLQ-C30 [56]	✓	✓	✓
		Melanoma-specific HRQoL	Functional Assessment of Cancer Treatment-Melanoma (FACT-M) [57]^f^	✓	✓	✓
		Self-rated health and economic evaluation	EQ-5D-5L [58]^g^	✓	✓	✓
		Anxiety and depression	Hospital Anxiety and Depression Scale (HADS) [59]^h^	✓		✓
		Immune-related adverse events	Immunotherapy-specific questionnaire (EORTC item bank)^i^	✓	✓	✓
		Information needs and satisfaction	EORTC QLQ-INFO25 [60]^j,k^	✓		✓
	**Usability**					
		User friendliness and usability	System Usability Scale (SUS) [61]		✓	
		Patient trust to health service	Patient Trust Assessment Tool (PATAT) [62]		✓	
		Quality of mobile health apps	User Version of the Mobile Application Rating Scale (uMARS) [63]			✓
	**Feasibility**					
		Recruitment rate	Percentage of patients included in the study out of the eligible patients	✓	✓	✓
		Patient compliance	Percentage of patients completing the questionnaires	✓	✓	✓
		Patient retention	Percentage of patients adhering to the app	✓	✓	✓

^a^CAPABLE: Cancer Patients Better Life Experience.

^b^PROM: patient-reported outcome measure.

^c^EORTC: European Organization for Research and Treatment of Cancer.

^d^QLQ-C30: Quality of Life Questionnaire-Core 30.

^e^HRQoL: health-related quality of life.

^f^Of the FACT-M, we use the Melanoma Subscale and the Melanoma Surgery Subscale, items specific to quality of life in patients with melanoma. High scores show a high quality of life. Testing has shown that the FACT-M is a reliable and valid instrument to assess quality of life in patients with melanoma.

^g^The EQ-5D is a standardized 5-level, 5-dimensional multi-attribute utility questionnaire that measures mobility, self-care, usual activities, pain/discomfort and anxiety/depression, using a five dimension scale.

^h^Psychological distress will be assessed with the Hospital Anxiety and Depression Scale (HADS). The HADS, a 14-item questionnaire, assesses symptoms of mood disturbance, yielding separate scale scores for anxiety and depression, as well as a total score.

^i^Immunotherapy-specific questionnaire. In assessing quality of life in patients with cancer, it is recommended to use a generic and cancer-specific measure of quality of life plus a treatment-specific questionnaire. However, to date the available validated measurements do not include the problems and symptoms of immunotherapy. Therefore, we identified, based on literature and expert opinion, 19 symptoms and created a symptom list based on items of the EORTC item Library.

^j^Fulfilment of information needs will be measured by the EORTC QLQ-INFO25 questionnaire. This validated 25-item questionnaire incorporates four information provision subscales: perceived receipt of information about the disease, medical tests, treatment and other care services.

^k^QLQ-INFO25: Quality of Life Questionnaire-Information 25.

### Sample Size Calculation

As this is an exploratory study, a rough sample size calculation was performed based on preliminary changes in fatigue in the control cohort and feasibility parameters. In the control cohort (currently composed of 70 patients with melanoma that started with ICIs), an increase of 15 points in fatigue is shown in the first 3 months of treatment (nonpublished data). When providing the CAPABLE system to patients in the pilot study, we expect significantly less worsening of patients’ fatigue levels (by 5 points less increase in fatigue). With a 10-point difference and an SD of 20, the selected effect size is 0.5. Since the inclusion period is restricted to 6 months due to project obligations, we use an enrollment ratio of 0.3 (two-third of the control cohort and one-third of the CAPABLE cohort). Together with a 2-sided α of .05 and an accepted power of 70%, the needed sample size would be 139 patients, of whom 107 will be enrolled in the control cohort and 32 will be enrolled in the CAPABLE pilot study. We anticipate a dropout rate of 5%-10% because of unforeseen rapidly progressive diseases that might occur in this patient population. Questionnaire dropouts are considered minimal because the research team will see the patient around their 3- and 6-month follow-up at the patient’s hospital appointments. A compliance rate of 60% to participate in and use the CAPABLE system is considered feasible and clinically acceptable, meaning 36 patients have to be included to meet this feasibility end point. Therefore, we need to have 60 eligible patients in the 6-month inclusion period.

### Statistical Methods

Descriptive statistics will be calculated to provide information about the patient population. The mean scores of fatigue and other questionnaire outcomes will be calculated using algorithms in the existing literature, and these scores will be used as end points for analyses. Summarizing and visualizing methods such as line graphs will be used to make the data more interpretable. Effect sizes will be calculated using standard statistical procedures. The difference in fatigue (improvement or worsening) over time (baseline and follow-up moments) will be analyzed either using a linear mixed model or generalized estimating equation analysis. We will adjust for baseline patient-reported outcome scores and other covariates such as sociodemographic variables, disease, and treatment characteristics. To compare the mean fatigue scores between the group receiving the CAPABLE intervention and the control group at each individual time point, we will use *t* tests or Mann-Whitney U tests. Patients will be individually matched on sex, age, tumor staging (stage III or IV), and treatment (mono- or combination therapy). For differences in fatigue between the groups over time, we will use repeated measures ANOVA. A *P* value of <.05 will be seen as statistically significant; however, according to Cocks et al [65], a mean difference in change scores (per subdomain) can be seen as clinically relevant even if this is not statistically significant. Therefore, statistical and clinically meaningful differences will be analyzed. However, as this is an exploratory study, analysis will mostly consist of descriptive statistics, as *P* values are expected to render most outcomes nonsignificant.

Missing items from the questionnaires will be imputed according to the corresponding guidelines. The scale scores of the EORTC QLQ-C30 will be set to missing if fewer than half of the items on a given scale are answered. Where at least 50% of the relevant scale scores will be present, the missing values can be replaced by the mean of the present values. Statistical analyses will be done using R (R Core Team) [66].

### Patient and Public Involvement

One of the CAPABLE consortium members is an Italian patient association that was involved in the development of the CAPABLE app. Furthermore, the development of CAPABLE went through multiple research phases. Patient and clinician interviews were held to establish baseline needs and wish for the CAPABLE system (P20CAP; IRBd20-085), of which a paper is published [43]. HCPs provided valuable input for the clinical validation of the implemented guidelines. Furthermore, multiple patients and HCPs provided their valuable input to enhance the quality of the system during the development of the CAPABLE app through multiple usability testing rounds (P21CAP; IRBd21-139). The results of the study will be communicated to participants if wish.

### Ethics Approval

Ethical approval was granted by the Medical Ethical Committee of NedMec (Amsterdam, The Netherlands) under reference number 22-981/NL81970.000.22. Any amendments will be reviewed by this Medical Ethical Committee. CAPABLE is registered as a medical device trial according to the Medical Device Regulation, article 62. The trial is prospectively registered at ClinicalTrials.gov (NCT05827289). Original informed consent signed by patients allows for the analysis of the primary and secondary outcomes of this study. In case additional data needs to be analyzed, additional informed consent will be asked. Subject data will be pseudonymized and key-coded. No compensation is provided to participating patients. This study is conducted in accordance with the principles of the Declaration of Helsinki, version 9, October 2013, and the Medical Research Involving Human Subjects Act (WMO), including International Organization for Standardization (ISO) 14155 (medical device use).

## Results

Inclusion in this trial started late in April 2023 and is currently enrolling. We expect to finalize inclusion by the end of August 2023. The expected last follow-up measurement will be collected in February 2024, after which data cleaning and data analysis will start. Preliminary data analysis will be carried out in December 2023 due to a project deliverable deadline by the end of December 2023.

## Discussion

In this pilot study, we will explore the effect of using the CAPABLE app immediately after melanoma diagnosis in 36 patients, providing symptom monitoring, education, and well-being interventions during their first phase of treatment with ICIs, on 3- and 6-month HRQoL outcomes such as fatigue and functioning, and the patient acceptability of using a system such as CAPABLE. Since treatment with ICIs is becoming standard therapy for patients with high-risk and advanced melanoma, more and more patients experience treatment-related AEs. One of the most common symptoms of this treatment is fatigue. Consequently, a decrease in short- and long-term HRQoL can occur in these patients. An approach to improving symptom control and HRQoL outcomes is to use an eHealth tool. eHealth apps may contribute to improving HRQoL by addressing the unmet supportive care and information needs of these patients by enabling autonomy and self-management. Moreover, eHealth tools have shown promising results in improving fatigue by stimulating physical exercise, psychoeducation, mindfulness-based interventions, and yoga. However, previous studies focused on eHealth apps providing either symptom monitoring or interventions targeting fatigue.

It is hypothesized that the CAPABLE intervention will limit the increase in fatigue in patients with melanoma receiving ICIs by 10 points at 3 months after the start of treatment compared to a similar patient group receiving standard care. Furthermore, it is hypothesized that the system will be feasible to implement during ICI treatment in clinical practice. This system will be evaluated by 36 patients for 3-6 months from the start of their ICI treatment. End points of the study will be measured at baseline, 3 months, and 6 months after the start of treatment.

Several limitations of this pilot study should be noted. First, the intervention period will be 3 to maximally 6 months due to the limited number of well-being interventions in the mobile app and information provision focusing on the first period of treatment. Therefore, we will not be able to evaluate the long-term effects of the CAPABLE mobile intervention. Second, the inclusion period will be restricted to 6 months, as the time frame of the study is limited by project restrictions and available funding. Third, the control group is collected before the intervention group (ie, the CAPABLE cohort). Because of time restrictions in the project and the limited number of patients available in the hospital, it was necessary to create 2 separate prospective cohorts instead of using a randomized design. Moreover, the control group was collected during the COVID-19 pandemic, and this might have negatively influenced the HRQoL outcomes. Furthermore, we chose the short EORTC QLQC30 fatigue scale to measure our primary outcome, as we already use this questionnaire in our daily clinical routine, facilitating future implementation. As it is short, it minimizes patient burden. Furthermore, it was previously shown that there was no difference in chronic fatigue as measured by the EORTC QLQ-C30 and the validated Fatigue Assessment Scale (FAS) in Hodgkin lymphoma survivors [67]. Lastly, this pilot study does not include the possible relevance of supportive care provided for patient’s informal caregivers. It is well-known that informal caregivers or relatives of patients with cancer experience high caregiver burden and distress [68]. eHealth interventions could offer a convenient approach for providing support to the informal caregivers of individuals facing cancer [69]. Future studies with the CAPABLE app should focus on including support for informal caregivers.

Nevertheless, this pilot trial has several strengths, including the prospective study design of the CAPABLE group and the control group. Furthermore, both study groups are collected from a population receiving standard care and do not participate in experimental trials. The CAPABLE smartphone app is developed following user-centered design principles and in close collaboration with patients, app developers, component developers, and technical and clinical staff. Initial prototypes were built after content-generating interviews with patients and HCPs, following 3 rounds of user experience and user interface testing in both target populations. Consequently, the content offered in the CAPABLE app is of high quality and reviewed by the local medical staff in collaboration with patients. Last, although not primarily focusing on evaluating the change in care structures, it is worth mentioning that an eHealth tool such as CAPABLE may lead to earlier intervention for immune-related AEs and psychological complaints. A future and larger trial should focus on the impact of the implementation of such tools and their effect on existing care structures.

In conclusion, the CAPABLE pilot study will provide preliminary evidence about the effect, usability, and feasibility of the CAPABLE system, which may encourage further studies with this eHealth app. A similar pilot trial with the same CAPABLE system but for another population is currently enrolling patients in an Italian hospital. These studies will be the first to provide a system containing symptom monitoring, information provision, and well-being interventions in one eHealth tool. When positive results are generated on the primary outcome, outcomes should be validated in a larger randomized controlled trial, keeping end points for possible implementation in mind. Adding the CAPABLE system to active treatment is hypothesized to decrease fatigue in patients with high risk and advanced melanoma during treatment with ICIs compared to a control group receiving standard care.

## Abbreviations

AE: adverse event
CAPABLE: Cancer Patients Better Life Experience
CRF: cancer-related fatigue
CTCAE: Common Terminology Criteria for Adverse Events
EHR: electronic health record
EORTC: The European Organization for Research and Treatment of Cancer
FAS: Fatigue Assessment Scale
GAD-7: Generalized Anxiety Disorder-7
HCP: health care professional
HRQoL: health-related quality of life
ICI: immune checkpoint inhibitor
ISO: International Organization for Standardization
NCI: National Cancer Institute
NKI: Netherlands Cancer Institute
PHQ-9: Patient Health Questionnaire-9
PRO-CTCAE: Patient-Reported Outcome version of the Common Terminology Criteria for Adverse Events
QLQ-C30: Quality of Life Questionnaire-Core 30
SUS: System Usability Scale
VC: Virtual Capsule
WMO: Medical Research Involving Human Subjects Act

## Data Availability

The data sets generated during or analyzed during this study are not publicly available due to the sensitive information of patients in this data set, but are available from the corresponding author on reasonable request.
